# Experimental Evidence of Classical Conditioning and Microscopic Engrams in an Electroconductive Material

**DOI:** 10.1371/journal.pone.0165269

**Published:** 2016-10-20

**Authors:** Nicolas Rouleau, Lukasz M. Karbowski, Michael A. Persinger

**Affiliations:** 1 Biomolecular Sciences Program, Laurentian University, Sudbury, Ontario, Canada; 2 Behavioural Neuroscience Programs, Laurentian University, Sudbury, Ontario, Canada; Waseda University, JAPAN

## Abstract

Synthetic experimental substrates are indispensable tools which can allow researchers to model biological processes non-invasively in three-dimensional space. In this study, we investigated the capacities of an electroconductive material whose properties converge upon those of the brain. An electrically conductive material composed of carbohydrates, proteins, fats, ions, water, and trace amounts of other organic compounds and minerals was classically conditioned as inferred by electrophysiological measurements. Spectral densities evoked during the display of a conditioned stimulus (CS) probe were strongly congruent with those displayed during the conditioned-unconditioned stimulus pairing (CS-UCS). The neutral stimulus consisted of the pulsed light from a LED. The unconditioned stimulus was an alternating current. Interstimulus intervals >130 ms did not result in conditioned responses. Microscopic analysis of the chemically-fixed substratum revealed 10–200 μm wide ‘vessel structures’ within samples exposed to a stimulus. Greater complexity (increased fractal dimensions) was clearly discernable by light microscopy for stained sections of fixed samples that had been conditioned compared to various controls. The denser pixels indicated greater concentration of stain and increased canalization. Implications for learning and memory formation are discussed.

## Introduction

In 1955, Cragg and Temperley [1] published a paper in the journal *Brain* which indicated that conditioned responses could be elicited from iron bars by applications of paired, steady-state (DC) and alternating electromagnetic fields (AC) which would operate as unconditioned-conditioned (UCS-CS) stimuli. Their theory offered a basis for the stability of neural engrams with the suggestion that ferromagnetic hysteresis models the history-dependent output functions of neural systems. The suggestion is not that neurons display ferromagnetic hysteresis. Rather, the observed stimulus-response patterns of the iron bar [1] are governed by the same basic operating principles as those observed in the nervous system. If the theory is robust and models a general learning principle independent from ferromagnetic hysteresis, the observed history-dependent output functions should apply to other non-biological substrates. As indicated by their original approach, the theory was not necessarily limited to living systems and would provide a general mechanism by which information could be encoded into organized materials as pair-associated events. Here, we present one type among what could be very many types of simple neural-like simulators which display microvolt fluctuations as potential differences with coupled microstructural reorganizations indicative of associative learning where outputs are dependent upon a history of inputs.

Associative learning is a feature of the nervous system that has been studied extensively [2,3] and represents one of many ways by which the microstructure of the brain can accommodate functional modifications. The storage, or more accurately, the representation and release or “retrieval” of information within brain-space as memory can be separated by great intervals of time. This property allows organisms to operate functionally beyond the specious present and to access the representation of events not accessible within the immediate sensory environment. Mechanisms underlying these processes have been characterized at the cellular and subcellular levels in biological organisms [4,5]. However, observations such as those presented by Cragg and Temperely [1] suggests a fundamental physical basis to learning which can perhaps be generalized to other systems. Nervous systems would therefore represent one of many types of systems that can store and process pair-associated information.

Classical conditioning is a type of associative learning which involves pairing stimuli in space-time (space-time contiguity) to elicit responses using previously neutral stimuli [6]. A stimulus must elicit a physiological response and necessarily be an event to which the organism is receptive. There are many forms of energy which can be sequestered by an organism. However, there are also innumerable environmental events that do not constitute stimuli at all. In classical conditioning, the neutral stimulus becomes a conditioned stimulus (CS) after being paired with an unconditioned stimulus (UCS). The presentation of the CS elicits a conditioned response (CR). The CR reflects major features of the UCR but is rarely identical to the UCR with respect to major properties, such as amplitude. The net effect of learning is that a previously neutral stimulus–generating no specific response–becomes capable of generating a response which it would not have generated if space-time contiguity of the CS and UCS had not occurred.

A variety of learning procedures have been developed which are collectively termed classical conditioning. Simultaneous conditioning involves presenting and terminating the CS and UCS during the same temporal interval [7]. The two stimuli therefore overlap entirely, requiring a narrow time-frame of reception on the part of the organism. Trace conditioning, however, involves presenting and terminating the neutral stimulus before the onset of the UCS [8]. A short interval of time devoid of any stimulation occurs between the two stimuli. This hiatus is traditionally labelled the “interstimulus” or trace interval. A represented trace of the neutral stimulus within the memory-encoding system is therefore required to effectively pair it with the UCS. This typically demands of the organism a capacity to store short-term traces of information for the minimum time of the interstimulus interval. Simultaneous conditioning is a classical method by which the learning capacity of an organism can be determined whereas trace conditioning represents a method by which one can determine its temporal range.

Recent work has demonstrated electrical activity characteristics, as inferred by measurements obtained using quantitative electroencephalography, of the neural correlates of consciousness in an electroconductive material designed to simulate a lesencephalic, simple cerebral cortex as is assumed in shell-models of electroencephalographic measurement. The presence of spectral profiles which overlap with those obtained from living human measurements was most conspicuous when the pH of the material was similar to the equivalent proton concentrations adjacent to the neuronal cell membrane [9]. The material displayed spectral densities within frequency bands characteristic of human brain activity, but only when the specific chemical and geometrical conditions were present. The most likely mechanism involved networks of interfacial water which would be critically important in modulating functions. The magnitude and the reliability of the results suggested that there could be information storage capacity for this material and that it might be able to “maintain the representation” of relevant stimuli. In other words, it might be “conditionable” and display the most fundamental operations of learning.

The aim of the current study was to induce a conditioned response in an electroconductive material whose properties exhibited the major chemical characteristics of brain tissue without the inclusion of cell bodies or any biological, modular nodes. The experimental model, if successful, would represent a potential substrate within which electrical abnormalities of the brain involving memory can be studied systematically in three-dimensional space. To this end, two primary experiments were designed. Experiment 1 involved exposing the electroconductive material to an electric shock (UCS) paired simultaneously with an LED (CS) as a pulse pattern (10Hz). After a temporal delay, a probe (CS) was displayed in order to elicit a potential CR. Electrical potentials were recorded throughout the trials using QEEG equipment. Experiment 2 involved pairing the same UCS and CS with inter-stimulus delays of varying durations in order to discern the temporal sensitivity of the material. A confirmed CS-CR pattern was operationalized as a display of spectral densities within a probe period (CS only) which significantly correlated with the spectral density profile of the UCS-UCR as measured by QEEG during the training phase. In other words, the electrical patterns displayed by the electroconductive material when it was being shocked should, in principle, have been displayed during the probe phase if the LED-shock pairing was effective. These same patterns should have not been present during the pre-training baseline. The presentation of the LED pattern alone, initially, did not elicit a UCR (p>.05) and was therefore classified as a neutral stimulus.

Samples of the electroconductive material, exposed to various combinations of the UCS and CS, then underwent chemical fixation, processing, and paraffin embedding procedures that we typically utilize for histological studies. After staining the sectioned material, quantification of internal microstructures was performed using light microscopy. The purpose of this structural analysis was to compliment the functional analysis of the CS-CR relationship using QEEG. Together, these measures demonstrated the structure-function relationships which are characteristic of learning except they occurred in a non-biological, electroconductive material.

## Materials and Methods

### Electroconductive Material

The content of the conductive material, which is effectively a dough (46% flour, 31% water, 15% proton donor, 7% salts, ~1% other) material through which current can be passed has been described elsewhere [9]. A bleached, all-purpose flour (73% carbohydrate, 14% protein, and 13% other including fat, organic compounds, or minerals by mass) was mixed with water, table salt, vegetable oil, and lemon juice (proton donor) as per an open-source recipe by Squishy Circuits which is typically used as a pedagogical tool to demonstrate basic principles of circuit formation. The precise volumes which constituted a “recipe” as outlined originally were 237 cc of water, 355 cc of flour, 133 cc of lemon juice, 59 cc of table salt, 15 cc of vegetable oil, and 2cc of food colouring. For the purposes of the current study, homogenized subsets of the batch recipe were sampled in discrete volumes and served as material substrates on which the experimental procedures were performed. We are unaware of any published analysis of this material’s physical properties. Rouleau & Persinger [9] reported that the material displayed microvolt fluctuations which expressed spectral densities that correlated with human brain activity as inferred by modified QEEG measurements. These patterns were only evident for the material when the pH of the precursor solution was <2.53. It was calculated that this pH was representative of the spatially discrete, extracellular environment immediately adjacent to the cell where a monolayer shell of protons extending out by a Bohr radius or 5.6 ∙ 10^−9^ cm maintains the resting membrane potential. For the purposes of the present experiments, only electroconductive material associated with a pH <2.53 was used. The authors proposed that the capacity for the material to express the most fundamental patterns associated with “memory” should be demonstrable. This prediction can be confirmed by identifying the equivalent of a conditioned response elicited by the material.

### QEEG

A Mitsar quanitative electroencephalography (QEEG) amplifier box was converted into a general electrophysiological measurement device and was employed to measure relative shifts in the electrical activity of the material during conditioning trials. The montage consisted of a single electrode (Cz) placed at the apex of the spherical mass, referenced to an average ((ref-x + ref-y)/2)) of activity measured at sensor locations placed over left and right lateral extremities of the mass. The sampling rate was set to 250Hz, with a 45–75Hz notch filter to exclude ambient electromagnetic noise.

Raw microvolt (μV) data were extracted in 5 second sections and spectral analyzed. Baseline, stimulation, and probe period spectral densities were correlated within the narrow bandwidths associated with the respective UR periodicities. Based upon measurements obtained during the training phase (CS-UCS), frequencies coinciding with the greatest spectral density peak were selected as the defined conditioned response. Employing this method, a selected spectral peak z>2.0 would be defined with cases for +/- 1Hz included. The narrow band data (9 cases per band) then underwent non-parametric correlational analysis where pre-exposure baseline, stimulation, and probe period spectral densities were simultaneously analyzed. A “hit” or confirmed conditioned response would be characterized by significant non-parametric correlations between narrow-band spectral densities during the CS-CR period and the CS-UCS period. Significant correlations between these periods would indicate that the electroconductive material is expressing patterned microvolt potential fluctuations characteristic of the period during which current is passed through the mass while it receives only light stimulation (in the absence of current application). The degree to which the narrow-band spectral profiles of the UCR and CR differ would characterize the phase-shift elicited by the material.

### Conditioning Apparatus and Stimuli

**Fig 1:**
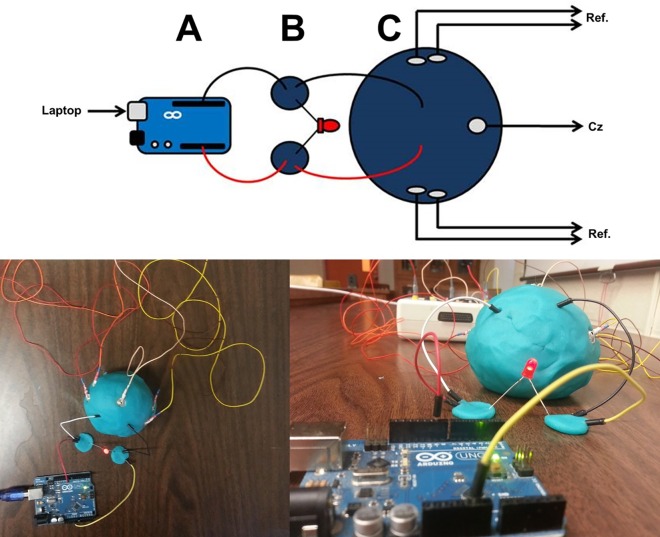
Conditioning apparatus. Stimuli are presented by a digital-to-analog microcontroller operating at 5V (A) into bilateral pieces of electroconductive material which holds an LED (B) in parallel and further still into the conditioning focus (C) which is referenced to its own lateral extremities (Ref) in a monopolar QEEG montage with the Cz sensor mounted at the apex of the spherical mass. The real experimental set up was photographed and is presented below the schematic.

The conditioning apparatus was constructed to manually administer stimuli with the capacity to systematically alter the interstimulus interval which separated the CS and UCS. An Arduino Uno R3 microcontroller coupled to an electronic breadboard received uploaded signal files via USB 2.0 from an HP ENVY laptop running Windows 8. Leads from the microcontroller ran current through a red light emitting diode (LED) placed 5 mm away from the electroconductive material. The LED had an associated emission wavelength of 620–625nm and an intensity of 150–200 millicandela. Direct leads to and from the material completed the circuit, allowing for simultaneous or lagged electrical shocks and light stimulation (see Fig 1). The program determined the temporal delay between the administration of the shock and the light stimulation. Once the files were uploaded, a reset button on the microcontroller was used to initiate the stimuli.

The unconditioned stimulus consisted of an all-or-none shock (1–5 V) administered directly as a current application into the electroconductive material. The lead, which was a standard electronic jumper cable, extended 0.5 cm into the electroconductive material. The duration of the direct stimulation varied contingent upon the procedure type described elsewhere.

### Procedure: Simultaneous Conditioning

**Fig 2:**
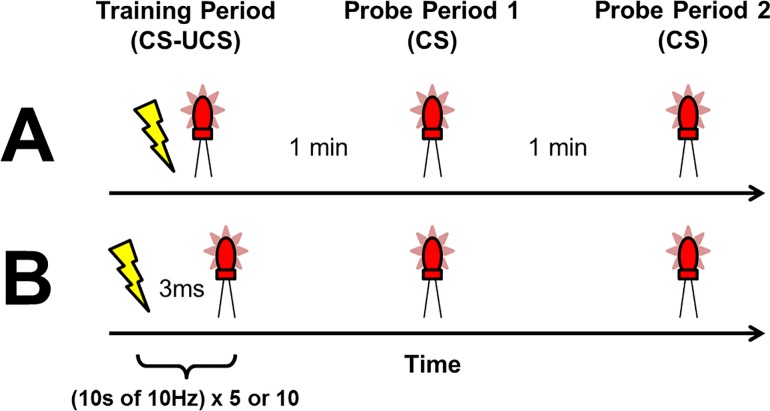
A comparative timeline of simultaneous (A) and trace (B) conditioning. Simultaneous conditioning during the training period is followed by a delay, which is 1 min in this example. A CS probe is then presented, followed by an additional delay and a final CS probe. The trace conditioning example involves an interstimulus interval between the CS and UCS during the training period, which is 3 ms in this example.

Four (n = 4) samples of electroconductive material, weighing approximately 70g each, were trained (CS-UCS pairing) and later exposed to a CS while being monitored for CRs as inferred by frequency-dependent electric potential differences. A total of twenty-four (n = 24) trials were conducted using the same samples. The training phase consisted of 5 or 10 brief, 10 second periods of 10Hz CS-UCS pairings presented sequentially and separated by 60 seconds. The simultaneously presented stimuli, with associated frequencies of 10Hz, are generated by presenting the LED and shock for 50ms with 50ms delays between each stimulus. Each cycle was therefore 100ms, which over 1000ms or 1s, gives 10 cycles of 10Hz. After the training phase, there was a short delay of 1 or 5 minutes followed by a probe (CS) in order to elicit a CR. This procedure is outlined in Fig 2A. QEEG recordings were obtained throughout all phases of the experiment.

All samples of the material were mechanically ablated after a given trial, recycling the material. This involved crushing the samples so as to remove any potential microstructures resultant of the treatment that might contribute to differences between the samples. In other words, the material samples were ‘reset’ for every new trial, but the material involved was not tested more than once within the same experimental condition in order to eliminate any potential redundant variance. μV^2^·Hz^-1^

### Procedure: Trace Conditioning

Four (n = 4) samples of the electroconductive material, weighing approximately 23g each, were trained and later exposed to a CS while being monitored for CRs. The training phases consisted of 5 brief 10 second periods of CS-UCS pairings where the interstimulus interval changed systematically across conditions. The duration of each LED or shock stimulus was 25ms. Nine (9) trace conditions were employed with the following interstimulus intervals: 3ms, 25ms, 100ms, 130ms, 150ms, 170ms, 200ms, 300ms, and 500ms. After the training phase and a 1 or 5 minute post-training delay, the samples were exposed to a probe (CS). This procedure is outlined in Fig 2B. QEEG recordings were obtained throughout all phases of the experiment. A total of 81 trials were completed. All samples were recycled using the method described for the simultaneous conditioning experiments.

### Histological Technique

Twenty (n = 20) 1 cm^3^ samples of the electroconductive material exposed to five conditions underwent histological processing. The first and second conditions involved exposure to simultaneous conditioning training protocols described elsewhere; however, the second condition was mechanically ablated. The third and fourth conditions involved exposures to light only and electrical shocks only, respectively. The fifth condition was that of a control wherein the samples were naïve to the training or any other protocol.

Samples were fixed in a solution of ethanol-formalin-acetic acid (EFA) for 7 days before systematic dehydration, and further chemically processed. The chemically-penetrated specimens were then embedded in paraffin wax blocks and sectioned into 10μm slices. Samples were then stained with Toluidine Blue O or Periodic Acid-Schiff and mounted.

### Microscopy Data Processing

Mounted samples of stained electroconductive material (n = 80) were photographed at 40x magnification with phase 2 contrast. A standardized method was used to capture the images whereby the upper left, upper right, and lower center extremities of the sample were photographed as internal measures of reliability, for a total of 240 images. Images were then uploaded to ImageJ and processed by Fractal Box Counting (FBC) and Colour Pixel Counter (CPC), methods which measure complexity as a function of scale and number of pixels of a given colour (e.g. red, blue, cyan, etc.). Whereas coloured Bitmaps (.bmp) were used for the CPC method, images undergoing FBC were converted to 8-bit format and grey-scaled before processing. To eliminate redundant variance, mean complexity and pixel measures were computed from the 3 images, reducing the cases to n = 80. Total pixels remained consistent across all images (~2∙10^6^ pixels).

## Results

### Experiment 1: Simultaneous Conditioning

**Fig 3:**
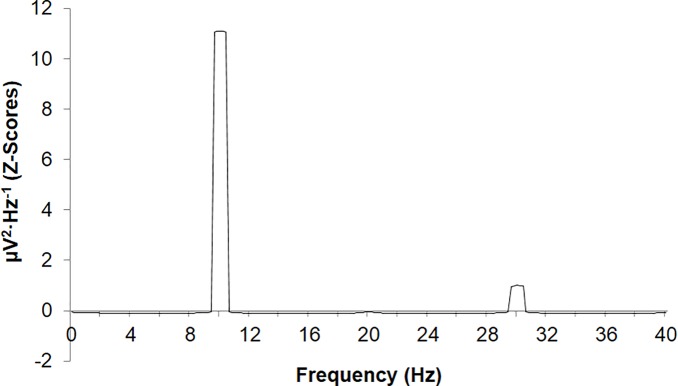
Mean standardized (z-score) spectral densities of the training phase (CS-UCS) displaying a clear peak (z>10) at approximately 10Hz. Harmonics are present at both 20Hz and 30Hz.

An examination of the spectral profile of the QEEG data associated with the stimulation period (UCS-UCR) during the training phase revealed a clear 10Hz peak which defines the CR (Fig 3). In other words, this is what the electroconductive material expresses as microvolt fluctuations when the shock (current application) is administered. Harmonics at 20Hz and 30Hz were noted with an emerging sub-harmonic at ~1Hz, confirming the UCS property of the 10Hz stimulus. All simultaneous conditioning experiments involved the 10Hz stimulus as the effective UCS-UCR. Consequently, the 9Hz to 11Hz band was selected as the window within which spectral densities across periods were correlated for simultaneous conditioning trials. Spectral densities within this band range associated with the electroconductive material exposed to the LED did not demonstrate 9Hz–11Hz spectral peaks with z > 2.0. In other words, the LED was determined to be a confirmed neutral stimulus as it did not elicit any particular response when presented alone.

Trials associated with a training phase consisting of five (5), 10s periods of 10Hz stimulation revealed significant correlations between spectral densities associated with electrical potentials recorded during the probe period (CR) and the training phase (CS-UCS) after a 5 minute delay immediately following the training phase, rho = -.71, p < .05. This relationship persisted after a 10 minute delay, rho = -.92, p < .001. Pre-training electrical potentials did not exhibit spectral content reflective of the UCS-UCR (p>.05). Ten (10), 10s periods of 10Hz CS-UCS pairings resulted in a significant correlation between spectral densities of electrical potentials during the training phase (CS-UCS) and probe period (CR) after a 5 minute delay following the training phase, rho = -.92, p < .001. However, spectral densities of electrical potentials obtained during the pre-exposure baseline also correlated with the probe period (CR) following a 5 minute delay post-training phase, rho = .80, p < .01. Together, these results suggest that although the electroconductive material expressed spectral characteristics of the CS-UCS, over-training or other temporal variables could have influenced the stimulus-response patterns.

### Experiment 2: Trace Conditioning

**Fig 4:**
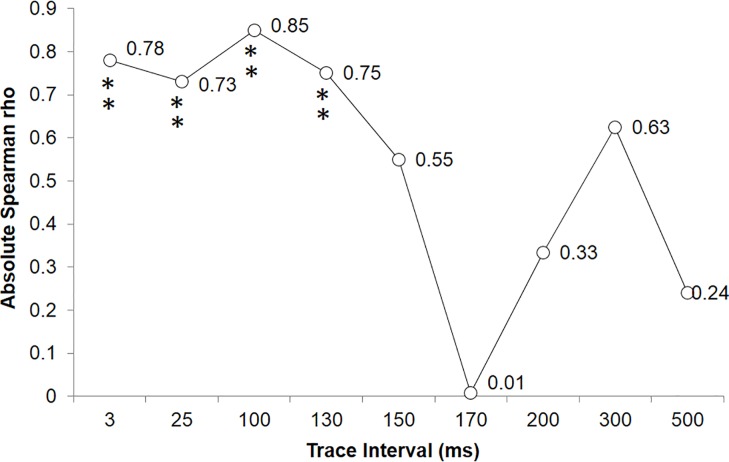
Plotted absolute non-parametric correlation coefficients (Spearman rho) demonstrating the systematic relationship between the spectral densities of the training phase (UCS-UCR) and the probe period (CS-CR).

A systematic analysis of potential CRs as a function of the duration of the interstimulus delay was revealing. Interstimulus intervals between 3ms and 500ms were examined (S7 File–S13 File). Probe period (CR) and training phase (CS-UCS) spectral densities were significantly correlated after a 1 minute delay following the training phase for the 3ms (rho = -.78, p < .05), 25ms (rho = .73, p < .05), 100ms (rho = .85, p < .005), and 130ms (rho = -.75, p < .05) interstimulus interval conditions. This was not the case for 150, 170, 200, 300, and 500ms conditions (p>.05). These results are represented in Fig 4.

**Fig 5:**
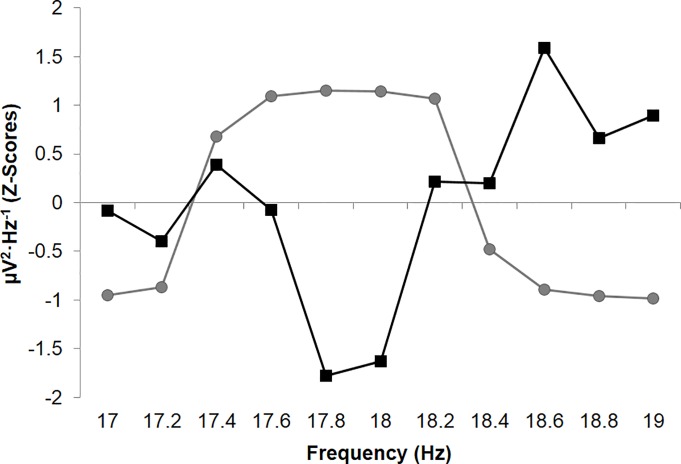
Mean standardized spectral densities (z-scores) computed from measurements obtained during the probe period (CR; squares) for the 3ms trace interval trials involving a 1 minute delay following the training phase with 0.2Hz increment deviations within the 17Hz to 19Hz band. Condition-matched data for the training phase or UCS-UCR (circles) is also plotted.

Fig 5 demonstrates the relationship between the CR and the CS-UCS for the 3ms interstimulus interval condition as standardized spectral densities, displaying approximately z = 2.0 deviations (with opposite direction) as a function of the frequency peak associated with the UCR within the 17Hz to 19Hz band (S6 File). This frequency band is relevant because the combined duration of the CS (25ms), UCS (25ms) and interstimulus delays (3ms) between the stimuli result in a cycle equivalent to 17.9Hz or ~18Hz. The true shift in raw μV values associated with the probe period was equivalent to a change of approximately 10 μV. As a relative comparison, the standardized shifts in spectral densities presented in Fig 3 associated with the training phase (for any trace condition) are equivalent to actual shifts of 1000–2500 μV.

Probe period (CR) and training phase (CS-UCS) spectral densities were significantly correlated after a 5 minute delay post-training phase for the 3ms (rho = -.85, p < .05), 25ms (rho = -.75, p < .05), 100ms (rho = .82, p < .005), 130ms (rho = -.68, p < .05), and 170ms (rho = -.68, p < .05) interstimulus interval conditions. This was not the case for 150, 200, 300, and 500ms conditions (p>.05). Pre-training spectral densities were also correlated with the training phase for the 130ms (rho = -.87, p < .005) and 170ms (rho = -.97, p < .001) interstimulus interval conditions. These results suggested an intrinsic property within the electroconductive material whereby an interval between the two events of less than 130 ms or the equivalent of 7.7 Hz could be associated. Intervals greater than 130 ms did not result in stimulus-response patterns characteristic of associative learning.

### Microscopy Data

**Fig 6:**
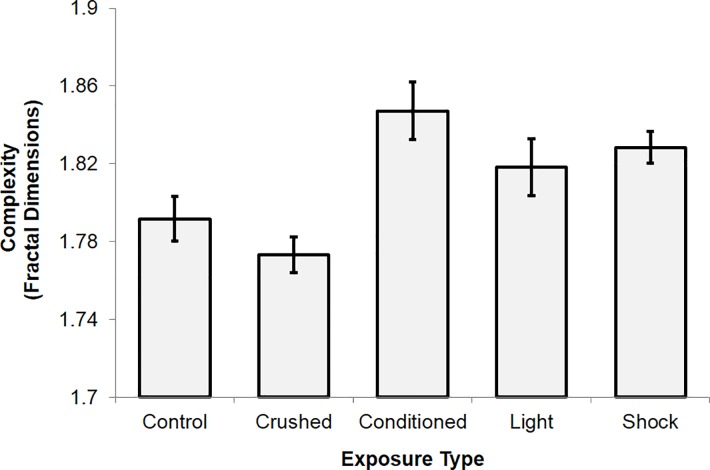
Mean complexity (fractal dimension) scores extracted from 8-bit, binary images of sectioned samples stained with TBO, plotted as a function of exposure type prior to chemical fixation. Standard error bars are provided.

Complexity measures for slides of paraffin-embedded sections of the fixed electroconductive material stained with Toluidine Blue O and Periodic Acid-Schiff were positively correlated (r = .53, p < .01, rho = .56, p < .005), demonstrating reliability of the tools. Only TBO-associated data (n = 40) are presented for simplicity, although it should be noted that effects were comparable for both stain types. An ANOVA revealed differences in complexity between exposure conditions, F(4,39) = 6.10, p = .001, explaining 41% of the variance (Fig 6). Conditioned electroconductive material displayed increased complexity relative to controls, t(14) = 2.99, p = .01, r^2^ = .39. Similarly, conditioned electroconductive material displayed increased complexity relative to those that had been crushed before being chemically fixed, t(14) = 4.29, p = .001, r^2^ = .57. Comparing complexity scores for controls and the conditioned-crushed group as well as controls and the light-exposed group revealed no significant differences (p>.05). However, shock-exposed electroconductive material was marginally more complex relative to controls, t(14) = 2.61, p < .05, explaining 30% of the variance. Complexity scores associated with light- and shock-exposed groups were significantly greater than those of the conditioned-crushed group, explaining 33% and 67% of the variance respectively. The conditioned, light-exposed, and shock-exposed groups displayed comparable levels of complexity (p>.05).

**Fig 7:**
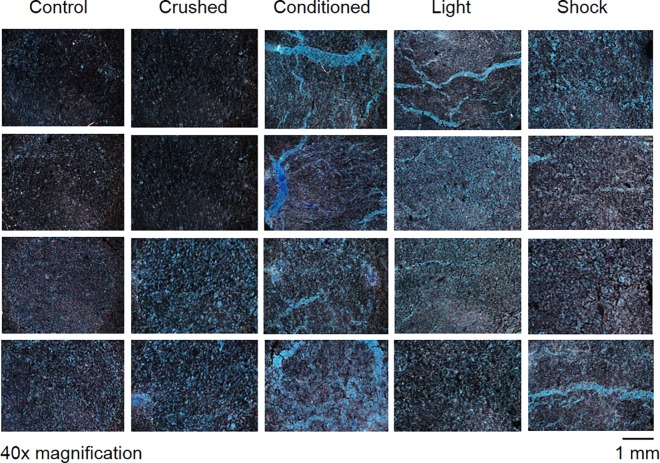
Samples stained with TBO, imaged at 40x magnification under Phase 2 contrast light microscopy classified by exposure type prior to chemical fixation. “Vessel structures” were measured to be 10–200 μm wide.

Statistically significant differences in the average number of blue pixels contained within photographs of stained slides (Fig 7), a measure of TBO staining, were observed as a function of the exposure conditions, F(4,39) = 3.38, p < .05, explaining 29% of the variance. A similar effect was noted for cyan pixels, F(4,39) = 6.91, p < .001, explaining 44% of the variance (S15 File). The conditioned group was associated with a greater number of cyan pixels than controls or the non-treated condition, t(14) = 2.36, p < .05, r^2^ = .28. The conditioned group was similarly associated with a greater number of cyan pixels relative to the conditioned-crushed group, t(14) = 2.61, p < .05, r^2^ = .33. Blue and cyan pixel counts were not significantly different when comparing control and conditioned-crushed conditions. However, controls were associated with less blue pixels relative to the light-exposed group, t(14) = -2.71, p < .05, r^2^ = .34. Controls were also associated with less cyan pixels relative to the light-exposed group, t(14) = -3.81, p < .005, r^2^ = .51. Differences were only obtained when examining cyan pixel counts when comparing controls to the shock-exposed group, t(14) = -2.96, p < .05, r^2^ = .38. The conditioned group, as well as light- and shock-exposed groups did not demonstrate any differences across blue or cyan pixel counts (p>.05).

**Fig 8:**
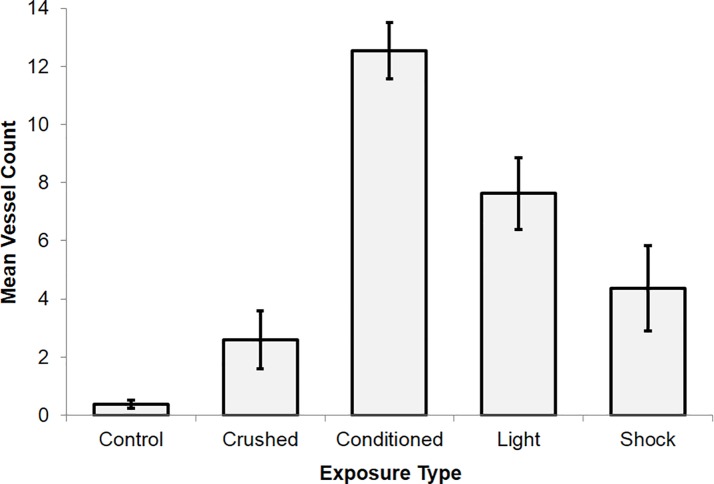
Mean vessel structure counts by exposure type for slides stained with TBO. Standard error bars are provided.

Three (n = 3) independent observers were asked to count the total number of ‘vessel structures’ for each slide (S14 File). Inter-rater measures indicated moderate to strong reliability. An ANOVA was then computed in order to identify any differences in total identified ‘vessel structures’ between conditions. A statistically significant difference was identified between the groups, F(4,117) = 20.90, p < .001, η^2^ = .43. Post hoc analyses indicated three distinct groups. The first consisted of Control (M = .38, SE = .15), Crushed (M = 2.58, SE = 1.00), and Shock (M = 4.36, SE = 1.46). The second group consisted of Shock (M = 4.36, SE = 1.46) and Light (M = 7.63, SE = 1.24). The third subset involved the Conditioned (M = 12.54, SE = .98) group alone. Post-hoc t-tests confirmed that the major source of variance was due to the significant differences between the control and crushed samples, the light without pairing with shock samples, and the conditioned samples. These results are presented in Fig 8.

## Discussion

The experiments resulted in some clear technical patterns which should be addressed. The increased representation of negative correlations between spectral densities associated with the CS-UCS period and the CS probe period requires further consideration. One potential explanation involves the method of information encoding. Negative-images or castings represent one method by which an object or information source can be effectively replicated and stored within a novel space. This is perhaps one of the simplest forms of information storage. In the case of negative-images, 0101 is stored as 1010 and, assuming maximum efficiency, the relationship between the original signal and stored information would be equivalent to a correlation coefficient of r = -1.0, rho = -1.0. Fig 5 demonstrates what might represent the relationship between the original signal and its stored counterpart, released upon display of a CS. Both signals center upon a common frequency with identical relative amplitude but opposite direction. This is the spectral-equivalent of a negative image.

There is evidence to suggest any stored information was localized rather than isotropically distributed. Consider the relationship between the primary sensor (Cz) and the references (Fig 1). An averaged reference over four (4) sensors, two of which are a means of grounding the system, provide a comparator by which potential differences are calculated over Cz. If electrical activity was homogeneous throughout the entire mass, the potential differences would be effectively or near zero. It was demonstrated that this was not the case. Therefore, any information detected as a signal was not being expressed everywhere simultaneously. If it were, potential differences would be zero and no effect would be discernible.

Although memory can be considered to involve complex processes for the representation of information within a brain or its simulated arrangements (the computer) the operation is much more universal. The symbolic representation of “memory” can be defined as an induction of some subset of Set A within Set B such that long after Set A is no longer adjacent or existent, that representation of Set A remains in Set B. This operation can be found in most of the traditional scientific disciplines and includes the concepts such as memory, genetic history, culture, and tradition. The microstructural changes within membranes and receptors of the cells that compose the immune system would be another classic example of “chemical memory” with referencing brain function.

Most experiments involving conditioning and learning in non-mammalian systems such as plants or inanimate objects such as the domains within iron exposed time-varying and static magnetic fields have employed direct measurements of discrete events. In the present study we employed the spectral power densities of the range of frequency variations within the special preparation of an electroconductive material to compare similarities between alterations from current induction only (the UCS), simply the presentation of the light from an LED, or the response to this light after it had been paired with the UCS. The fact that the “conditioned” group displayed a power spectral density that was most similar to the SPD elicited by electric current only is consistent with learning.

The congruence with the “conditioned” pattern of “power” density for the distributions of background potential differences in the electroconductive material and the “unconditioned” pattern of power densities due to the electrical stimulation suggests that comparisons of the temporal structures or the “field” properties of conditioned and unconditioned stimuli could add additional information to what lays at the bases of association through space-time contiguity. That this effect was not an artefact was indicated by the absence of this marked congruence of SPDs in response to the light only in electroconductive material if the delay between the CS and UCS had been more than 130 ms.

Solving for frequency or f = 1/T, the lower limit frequency of stimulus presentation which resulted in a paired association was 7.7Hz. This could suggest that implicit variations between 333 Hz to 7.7 Hz are an optimal range to effectively pair the CS-UCS in this pH-brain simulated electroconductive material. It could be relevant that the operating frequency of the hippocampus, a deep temporal lobe structure critically involved in mammalian trace conditioning [8], is the theta (4 to 7 Hz) range and particularly ~8Hz [10]. Temporal lobe atrophy and associated memory deficits are linked to the emergence of increased sub-theta (<7Hz) neural activity as inferred by QEEG [11]. This convergence upon intrinsic patterns of neural activity associated with learning in the mammal is supportive of a fundamental principle of temporal architecture of learning based upon contiguity which might not be restricted to living systems.

The structural changes within the electroconductive material in response to conditioning that were evident with classical histological methods suggest there was a physical correlate to the changes in SPD. These “engrams” were clear structural features of CS-UCS training group. Complexity of the material was highest when exposed to a stimulus, and lowest when unexposed or when mechanically ablated. Light and shock, both independently and in combination, produced 10–200 μm ‘vessel structures’. However, only after being paired with the UCS (AC current; shock) did the neutral stimulus (LED; light) display the definition of a CS by eliciting a CR In other words, stimuli with a history of space-time contiguity with a separate stimulus impinging upon the mass was sufficient to alter the internal structure in a different manner than if the stimuli had never been associated.

The range of width associated with these structures is within the typical diameter of most human cells. These microstructures were accompanied by increased blue and cyan pixel counts, indicative of increased stain uptake. Toluidine Blue is an acidophilic stain with a high affinity for nucleic acids [12], and is thought to heavily penetrate malignant epithelial tissue due to widening of intracellular canals [13]. We do not know if these structures are indicative of accreted molecules or increased spaces within the organizational matrix. However boundary conditions were formed that could serve as the physical correlate or even the “substrate” for the CR to simple light flashes but only after the conditioning had been completed.

What is clear is that significant energy was required within the mixture of constituents that comprised the electroconductive material to produce such visible obvious changes. Direct measurements indicated a potential difference of 50 mV across the electroconductive masses. The resistance was 15 kΩ and the electric stimulus (UCS) was between 1000 and 2500 μV. Hence the passive current would have been about 1.6 x 10^−7^ A. The equivalent across 5 cm would be 0.3 x 10^−5^ A/m. The equivalent voltage would be 0.5 x 10^−1^ V/m resulting in 0.15 x 10^−6^ W per m^2^. The CS (light) was 43 Lux (or 5 x 10^−2^ W per m^2^) on the surface of the material. Considering a conservative estimate of attenuation of a factor of 100 to 1000 of the incident light within the deeper regions of the material, there would a possible convergence of flux density within the constituents for both the light and the electric current. The estimates of capacitance from the physical dimensions of the “vessel structures” could be associated with capacitance ranging between 1·10^−6^ F per cm^2^ (10μm) and 2.8·10^−8^ F cm^2^ (200μm). If the resistance was about 3.75 x 10^5^ Ω cm^2^ the time constant would register between 10 and 40 ms, which are within the range of optimal duration for pairing of the CS and UCS for the material. If this concept is applicable to cerebral function then the relationship between the theta rhythms, the 8 Hz pulsatile cells within the second layer of the entorhinal cortices and the persistent 1 μm width of a synaptic cleft, width of a node of Ranvier, and typical interspine distance on a dendritic shaft may share a physical source.

In summary, the data indicate that a conditioned response can be encoded into a simple material, that the conditioned response is associated with structural modifications within the substrate, and that the structural features can be ablated by mechanical applications. Additionally, we have provided evidence which suggests that time constants intrinsic to the material might mediate the temporal window associated with the acquisition of the response as displayed by the material. The results indicate that non-neuronal materials could be used to model normal and abnormal electrical neural processes.

## Supporting Information

S1 File Simultaneous, 5 pairs, 1. Simultaneous conditioning with 5 pairs, data file 1.
(SAV)

S2 File Simultaneous, 5 pairs, 2. Simultaneous conditioning with 5 pairs, data file 2.
(SAV)

S3 File Simultaneous, 5 pairs, 3. Simultaneous conditioning with 5 pairs, data file 3.
(SAV)

S4 File Simultaneous, 10 pairs, 1. Simultaneous conditioning with 10 pairs, data file 1.
(SAV)

S5 File Simultaneous, 10 pairs, 2. Simultaneous conditioning with 10 pairs, data file 2.
(SAV)

S6 File Phase Graphing Data. Fig 5 standardized spectral data extracted.
(SAV)

S7 File Trace 100ms. 100 ms trace conditioning data file.
(SAV)

S8 File Trace 130ms. 130 ms trace conditioning data file.
(SAV)

S9 File Trace 150ms. 150 ms trace conditioning data file.
(SAV)

S10 File Trace 170ms. 170 ms trace conditioning data file.
(SAV)

S11 File Trace 200ms. 200 ms trace conditioning data file.
(SAV)

S12 File Trace 300ms. 300 ms trace conditioning data file.
(SAV)

S13 File Trace 500ms. 500 ms trace conditioning data file.
(SAV)

S14 File Vessel Counting. Vessel counting data file for histological slides.
(SAV)

S15 File Pixel Counting. Pixel counting data file for histological slides.
(SAV)

## References

[1] Cragg BG, Temperley HNV. Memory: The analogy with ferromagnetic hysteresis. Brain 1955, 78(2): 304–316. 13239912 10.1093/brain/78.2.304

[2] Pavlov IP. Conditioned reflexes: An investigation of the physiological activity of the cerebral cortex In Anrep G.V., Trans (Ed.). Dover Publications, Inc., New York 1927 10.5214/ans.0972-7531.1017309 PMC4116985 25205891

[3] Skinner BF. The behavior of organisms: An experimental analysis. Appleton-Century-Crofts, New York, 1938.

[4] Hawkins RD, Abrams TW, Carew TJ, Kandel ER. A cellular mechanism of classical conditioning in Aplysia: activity-dependent amplification of presynaptic facilitation. Science 1983, 219(4583): 400–405. 6294833 10.1126/science.6294833

[5] Malenka RC, Bear MF. LTP and LTD: An embarrassment of riches. Neuron 2004, 44(1): 5–21. 10.1016/j.neuron.2004.09.012 15450156

[6] Gormezano I, Kehoe EJ, Marshall BS. Twenty years of classical conditioning with the rabbit. Progress in Psychobiology and Physiological Psychology 1983, 10: 197–275.

[7] Burkhardt PE, Ayres JJ. CS and US duration effects in one-trial simultaneous fear conditioning as assessed by conditioned suppression of licking in rats. Animal Learning & Behavior 1978, 6(2): 225–230.

[8] Beylin AV, Gandhi CC, Wood GE, Talk AC, Matzel LD, Shors TJ. The role of the hippocampus in trace conditioning: temporal discontinuity or task difficulty?. Neurobiology of Learning and Memory 2001, 76(3): 447–461. 10.1006/nlme.2001.4039 11726247

[9] Rouleau N, Persinger MA. Cerebral Networks of Interfacial Water: Analogues of the Neural Correlates of Consciousness in a Synthetic Three-Shell Realistic Head Model. Journal of Signal and Information Processing 2014, 5(4): 143–154.

[10] Fries P, Nikolić D, Singer W. The gamma cycle. Trends in Neurosciences 2007, 30(7): 309–316. 10.1016/j.tins.2007.05.005 17555828

[11] Gambardella A, Gotman J, Cendes F, Andermann F. Focal intermittent delta activity in patients with mesiotemporal atrophy: a reliable marker of the epileptogenic focus. Epilepsia 1995, 36(2): 122–129. 7821268 10.1111/j.1528-1157.1995.tb00970.x

[12] Sridharan G, Shankar AA. Toluidine blue: A review of its chemistry and clinical utility. Journal of Oral and Maxillofacial Pathology 2012, 16(2): 251 10.4103/0973-029X.99081 22923899 PMC3424943

[13] Epstein JB, Scully C, Spinelli J. Toluidine blue and Lugol's iodine application in the assessment of oral malignant disease and lesions at risk of malignancy. Journal of Oral Pathology & Medicine 1992, 21(4), 160–163. 1376363 10.1111/j.1600-0714.1992.tb00094.x

